# Cropland mask dataset for the Canadian Prairies derived from Google satellite embedding imagery

**DOI:** 10.1016/j.dib.2026.112946

**Published:** 2026-06-06

**Authors:** Thuan Ha, Kwabena Abrefa Nketia, Shawn Neudorf, Steve J. Shirtliffe

**Affiliations:** a Department of Plant Sciences, College of Agriculture and Bioresources, University of Saskatchewan, Saskatoon, SK S7N 5A8, Canada; b Nutiren Digital Agriculture Centre, University of Saskatchewan, Saskatoon, SK S7N 5A8, Canada

**Keywords:** Agriculture, AlphaEarth, Remote sensing, Land-cover classification, Cropland, Random forest

## Abstract

This article presents a spatially explicit persistent cropland mask for the Canadian prairies covering Alberta, Saskatchewan, Manitoba. Data was generated using 64-band embedding data from AlphaEarth with Agri-Food Canada (AAFC) Annual Crop Inventory used for label generation. Stratified-random points from across the prairies were used with a Random Forest classifier to determine cropland and noncropland areas. The trained models were applied across the prairies from 2017 to 2024 in an annual wall-to-wall classification framework at 10 m resolution for each year. Annual classifications were then combined to create a multi-year frequency layer where pixels with more than two years of continuous cropping were labelled as cropland, creating a stable mask layer. The dataset contains a 10 m resolution binary raster mask layer in GeoTIFF format to support a wide range of applications including cropland mapping, land-use change assessment, agricultural monitoring, yield modelling, soil and climate studies, and machine-learning-based geospatial applications.

• Annual Prairie-wide cropland mask cloud-optimized GeoTIFFs generated using AlphaEarth data embeddings and Random Forest models trained on stratified random reference samples.

• Multi-year cropland frequency and stable cropland mask layers derived from aggregated annual predictions, enabling consistent identification of persistent cropland across the Canadian Prairies.

Specifications Table

**Table:** 

Subject	Earth & Environmental Sciences
Specific subject area	Cropland mask dataset for the Canadian Prairies derived from Google Satellite Embedding imagery.
Type of data	Raster (GeoTIFF) geospatial data
Data collection	The data was generated using supervised a machine-learning workflow where reference labels were derived from the AAFC Annual Crop Inventory. Stratified random points from across the prairies were used to extract predictor variables from the AlphaEarth data embeddings. The labelled data was then used to train Random Forest models and applied wall-to-wall to produce annual cropland predictions rasters for further aggregation and analysis.
Data source location	The data were generated for the Canadian Prairies (Alberta, Saskatchewan, and Manitoba), which cover the major agricultural regions of the study area between approximately 49°–55° N latitude and 96°–114° W longitude.
Data accessibility	Repository name: Crop mask of the Canadian Prairies)Direct URL to data: 1. Data published in Mendeley: link 2. Google Earth Engine (GEE) app for displaying the data: link 3. GEE code for accessing the data: link The dataset is publicly accessible through GEE. Users must have a Google account registered with GEE to access the assets. The data is openly available to all users with an active GEE account and can be accessed using a Gmail address associated with that account. All data can be visualized, queried, and exported directly within the GEE platform.
Related research article	None

## 1 Value of the Data

• The prairie-wide mask of active cropland is an excellent tool for researchers, private industry and governments when working with agricultural areas in Alberta, Saskatchewan, and Manitoba as the dataset provides an accurate raster map of active cropland that has excellent utility for a broad range of scientific fields beyond just agriculture including environmental, geographic, and economic studies.
• Any study that wishes to isolate cropland whether it is for analysis or comparison can make use of this dataset to isolate areas that have accurately been determined to contain cropland, for example a previous study of ours detecting field borders utilized a basic cropland mask as part of our workflow but the noise and lower resolution was one of the limiting factors to the accuracy of our field borders.
• Offers standardized GeoTIFF outputs compatible with widely used GIS platforms, cloud-based workflows, and high-performance computing environments for easy integration with remote sensing, climate, soil, and socio-economic datasets for spatial modeling and decision-support applications.

## 2 Background

Accurate high resolution cropland mapping is a foundational tool for agricultural and environmental monitoring, assessment and decision-making in the Canadian Prairies. AAFC Annual Crop Inventory [1] is a reliable dataset commonly used in both environmental and agricultural studies. However, at a 30 m spatial resolution it poses some accuracy limitations on field scale analyses and any other work requiring precise cropland detection. Despite widely available multispectral imagery and processing tools for 10 m resolution a 10 m cropland mask is still unavailable. This means that pixel level inaccuracies can propagate downstream through workflows and cause additional uncertainty. The continuous growth in cloud geospatial platforms such as GEE and advancements in machine learning methods have made large-scale high-resolution land cover analysis and mapping more feasible and reproduceable at scale. The demand for higher spatial resolution data is increasing for modern agro-environmental workflows and downstream tasks including crop classification, yield prediction, weed mapping, greenhouse gas and nutrient modelling, and land-use change assessments all stand have significant gains from high resolution cropland masking. It is worth noting that our previous study focusing on field border delineation using the Segment Anything Model used seasonal Sentinel-2 RGB composites with three visible bands [8,9]. The AAFC crop map was used to create cropland mask as a part of data processing but this study instead only uses that mask layer for training and instead uses the 64-dimensional AlphaEarth embedding dataset and multi year aggregation to create its cropland mask.

## 3 Data Description

The dataset is stored as tiled raster outputs generated on a regular spatial grid covering the Canadian Prairies. Cropland mask predictions were produced and exported on a per-tile basis to provide efficient and scalable access. Each tile has a unique grid identifier and contains aggregated multiyear cropland information derived from annual cropland predictions.

Tile-based outputs are organized by province within the GEE asset repository under the root directory projects/cropmask-alphearth/assets, within the CropMask folder. Raster filenames encode the temporal coverage (2017–2024), provincial identifier, and grid ID, following the naming convention:

(1) CropSum<startYear>_<endYear>_<*Province*>_gridID_<ID>

For example, a cropland mask tile for Alberta is stored as: projects/cropmask-alphearth/assets/CropMask/CropSum2017_2024_AB_gridID_1.

All tiles share a consistent 10 m spatial resolution and common spatial reference system for seamless mosaicking. Dataset access is openly available and requires a Gmail address associated with a GEE account.

## 4 Experimental Design, Materials and Methods

### 4.1 Data location

**Fig. 1:**
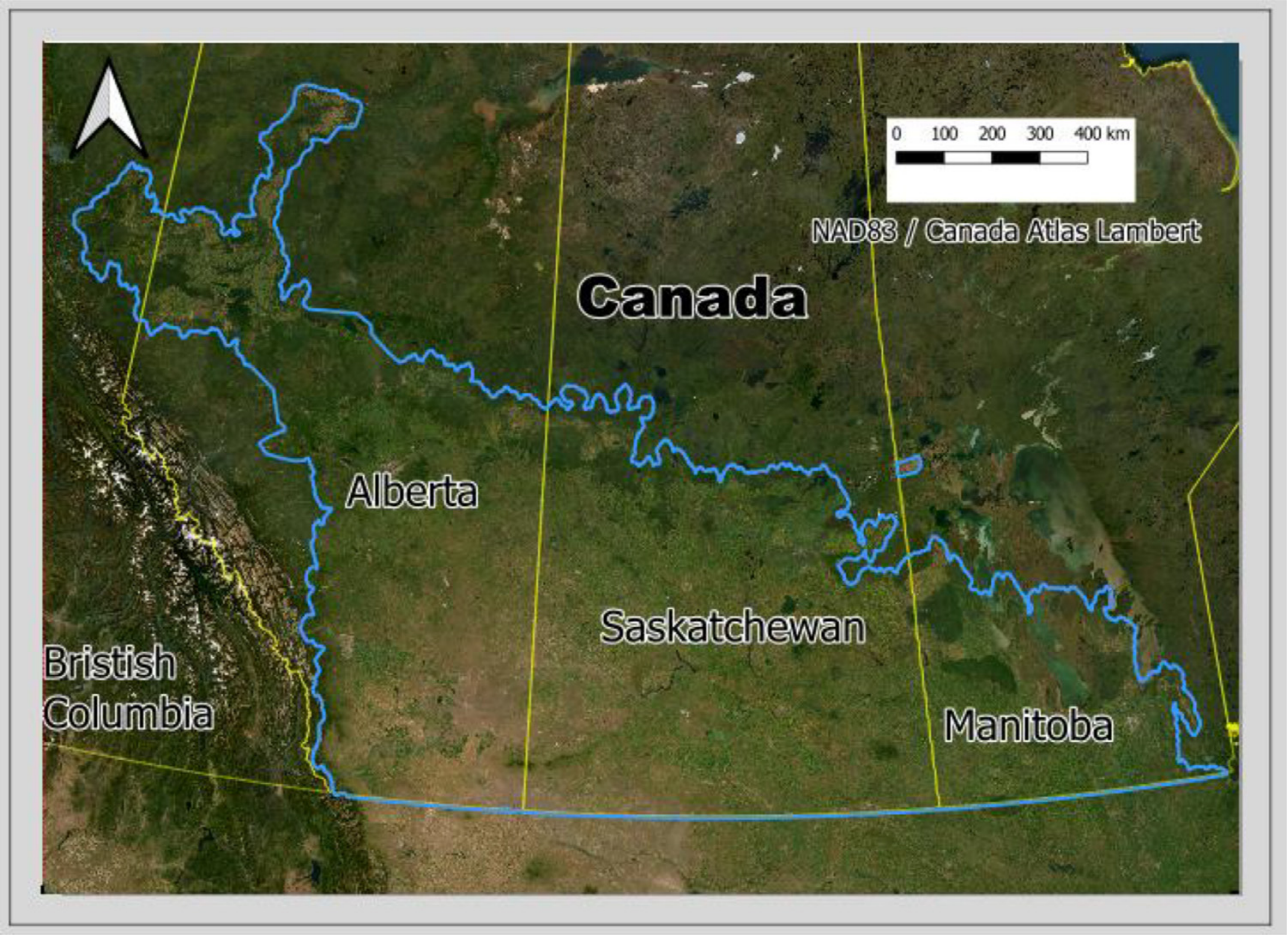
Data location showing agricultural regions of the Canadian Prairies (Alberta, Saskatchewan, and Manitoba).

The dataset covers the major agricultural regions of the Canadian Prairies, including Alberta, Saskatchewan, and Manitoba. The spatial extent spans approximately 49°–55° N latitude and 96°–114° W longitude (Fig. 1). The GeoTIFF map layers are stored and maintained as Google Earth Engine assets and are publicly available through the data repository accompanying this article.

### 4.2 Data sources

#### 4.2.1 AAFC annual crop inventory

Reference cropland information was obtained from the annual crop inventory produced by AAFC[1]. The annual crop inventory is generated using optical imagery and supervised classification at a spatial resolution of 30 m. The crop type includes standardized class definitions, quality control procedures, and multi-year temporal coverage, making it a reliable reference source for large-area agricultural studies. The labels cover a wide range of crop types and land uses but for the purposes of this study all agricultural classes were grouped into cropland and remaining classes as non-cropland. This approach provided a consistent and authoritative binary training reference for use with higher-resolution predictor variables.

#### 4.2.2 AlphaEarth Google satellite Embeddings

Predictor variables were derived from the Google Satellite Embedding dataset generated by the AlphaEarth Foundation mode [2]. This dataset is a global analysis ready set of geospatial embeddings derived from multi-source Earth observation data on the GEE platform [3]. At a 10 m spatial resolution Each pixel is represented by a 64-dimensional embedding vector that encodes annual temporal trajectories of surface conditions from a range of inputs including optical, radar, lidar and other sensors alongside texture information [4]. The embeddings go beyond conventional spectral bands or indices and summarize multi-modal relationships in compact feature space. The embeddings are unit-length, linearly composable, and robust to common data quality issues such as cloud contamination, sensor artifacts, and missing observations. These properties make them well-suited for large-scale classification, regression, and change-detection tasks. The AlphaEarth embeddings provide temporally aligned and information-rich predictor variables that can be directly integrated into scalable, cloud-based geospatial workflows [[5], [6], [7]].

#### 4.2.3 Sample collection

Training and validation samples were created by pairing AAFC-derived labels with predictors extracted from AlphaEarth embeddings. The AAFC map was reclassified into a binary mask by grouping all agricultural crop classes into cropland and retaining non-agricultural labels as non-cropland. Spatially stratified sampling from all three provinces was used for representative coverage across the prairies, covering a variety of agro-ecological zones and landscapes. Samples near class boundaries and areas containing mixed pixels were excluded where possible to reduce label noise and autocorrelation, with multi-year sampling was then used to capture interannual variability in cropping patterns and surface conditions. The final sample set was then used for both training and evaluating the model.

#### 4.2.4 Model training and prediction

**Fig. 2:**
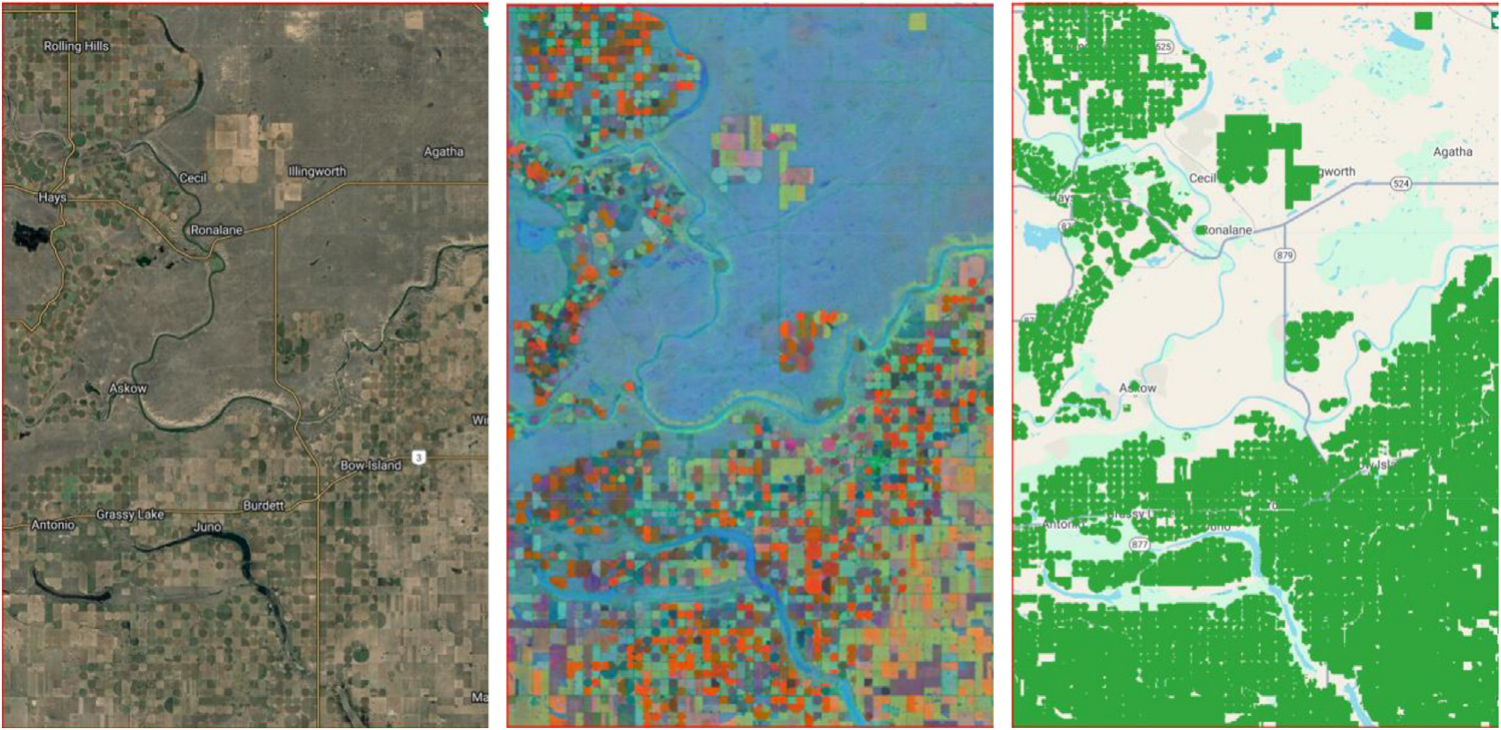
Subset maps showing the base map (left), AlphaEarth predictor data (middle), and the derived crop land mask (right) for a representative area in west Medicine Hat, Alberta, Canada.

The cropland classification models were trained using a Random Forest algorithm implemented in GEE. Training samples consisted of the binary cropland samples derived from the 30 m AAFC annual crop inventory map and the corresponding predictor variables from the AlphaEarth satellite embeddings. The model was trained on spatially distributed samples to support robust generalization across diverse agro-ecological conditions in the Canadian Prairies. The Random Forest model was configured using standard ensemble parameters. Once trained, it was saved as a reusable Earth Engine classifier and applied to annual AlphaEarth embedding imagery to generate cropland probability predictions at 10 m spatial resolution. Final cropland predictions were made by aggregating multiple years of predicted masks from 2017 to 2024 and creating a prediction probability for active cropland. Cropland found to be active for more than two years of continuous cropping was then classified as active cropland in the final mask layer. Model inference and large-scale batch prediction were subsequently automated using the Earth Engine Python API, enabling tile-based processing, export of prediction results, and efficient download of raster outputs for downstream analysis and archival. Fig. 2 presents an example subset of input and output maps.

#### 4.2.5 Dataset assembly

All input datasets and model outputs were assembled within the GEE environment. Tile-based cropland mask outputs were mosaicked by province and across the study area for visualization and quality assessment and consistency checks. Assembled outputs were displayed and inspected using custom scripts in the GEE Code Editor and made publicly accessible through an interactive GEE web application, allowing users to visualize Prairie-wide cropland masks and explore spatial patterns across regions and years.

#### 4.2.6 Software and tools

The dataset was generated using a reproducible computational workflow that integrates cloud-optimized geospatial processing and visualization. The software tools, libraries, and code resources used to generate and disseminate the dataset are listed below to support transparency and reuse.

• The GEE was used for large-scale data processing, mosaicking, visualization, and quality control. Final outputs can be explored without a GEE account through a public web application (link: GEE Apps)
• Interactive visualization of assembled outputs and tiled mosaics is available through the GEE Code Editor using a custom visualization script (link: GEE code)
• Code availability: All scripts used for data preprocessing, model inference, tile-based export, aggregation, and dataset assembly are publicly available in the associated GitHub repository link:

– https://github.com/thuanhavan/Prairies-Crop-Land-Mask-using-AAFC-and-AlphaEartth-data
– https://github.com/acilabuofs/Prairies-Crop-Land-Mask-using-AAFC-and-AlphaEartth-data

All code required to reproduce the dataset generation workflow is provided in the accompanying repository to facilitate reuse, adaptation, and extension of the dataset for future research and applications.

## Limitations

This cropland mask does have some limitations because the model is dependent on AlphaEarth predictor availability, quality and temporal consistency. Input gaps, inconsistent or inaccurate data can affect the predictions for certain regions and years. It should also be noted that the multi-year data aggregation reduces but does not eliminate potential salt and pepper artefacts, with there likely being some remaining noise in heterogeneous landscapes and along boundaries. It is still recommended for the user to implement some kind of post processing, spatial filtering or object-based smoothing when using the product for fine scale or field level applications.

## Ethics Statement

None.

## CRediT Author Statement

**Thuan Ha:** Conceptualization, Methodology, Data processing, Software, Writing- Original draft preparation. **Kwabena Abrefa Nketia, Shawn Neudorf:** Software, Methodology, Validity tests, Data curation, Writing- Reviewing and Editing. **Steve J. Shirtliffe:** Supervision, Funding acquisition, Writing- Reviewing and Editing.

## Data Availability

(Earth/Chem).Praires crop mask (Original data)

## References

[1] Agriculture & Agri-Food-Canada, Annual Crop Inventory 2024, 2025, Government of Canada Open Government Portal.

[2] Google-DeepMind, Satellite embedding V1, 2025, Earth Engine data catalog.

[3] Amani M. (2020). Google Earth Engine Cloud computing platform for remote sensing big data applications: a comprehensive review. IEEE J. Sel. Top. Appl. Earth. Obs. Remote Sens..

[4] Pasquarella V., Schechter E. (2025). AI-powered pixels: introducing Google’s Satellite embedding dataset. Google Earth Earth Engin..

[5] Yang L. (2026). Evaluating the performance of AlphaEarth Foundation embeddings for irrigated cropland mapping across regions and years. Remote Sens..

[6] Y., Cheng, Q. Zhu, and L. Fan, From landslide conditioning factors to satellite embeddings: evaluating the utilisation of Google AlphaEarth for landslide susceptibility mapping using Deep Learning. arXiv preprint arXiv:2601.07268, 2026.

[7] C.F., Brown, et al., Alphaearth foundations: an embedding field model for accurate and efficient global mapping from sparse label data. arXiv preprint arXiv:2507.22291, 2025.

[8] Ha T. (2026). A field boundary dataset for the canadian prairies derived from sentinel-2 imagery using the segment anything model. Data Br..

[9] Ha T. (2026). Field boundary delineation with seasonal sentinel 2 imagery using segment anything model (SAM). MethodsX.

